# Exploring the role of osteoglycin in type 2 diabetes: implications for insulin resistance and vascular pathophysiology

**DOI:** 10.1152/ajpendo.00320.2023

**Published:** 2023-10-11

**Authors:** Sheila González-Salvatierra, Beatriz García-Fontana, Luis Martínez-Heredia, Jesus Lacal, Francisco Andújar-Vera, Raquel Sanabria-de la Torre, Enrique Moratalla-Aranda, Silvia Lozano-Alonso, Cristina García-Fontana, Manuel Muñoz-Torres

**Affiliations:** ^1^Biosanitary Research Institute of Granada (ibs.GRANADA), Granada, Spain; ^2^Endocrinology and Nutrition Unit, Hospital Universitario Clínico San Cecilio, Granada, Spain; ^3^Department of Biochemistry and Molecular Biology II, University of Granada, Granada, Spain; ^4^CIBER of Frailty and Healthy Aging (CIBERFES) Institute of Health Carlos III, Madrid, Spain; ^5^Department of Cell Biology, University of Granada, Granada, Spain; ^6^Laboratory of Functional Genetics of Rare Diseases, Department of Microbiology and Genetics, University of Salamanca (USAL), Salamanca, Spain; ^7^Institute of Biomedical Research of Salamanca (IBSAL), Salamanca, Spain; ^8^Bioinformatic Research Service, Biosanitary Research Institute of Granada (ibs.GRANADA), Granada, Spain; ^9^Department of Computer Science and Artificial Intelligence, University of Granada, Granada, Spain; ^10^Andalusian Research Institute in Data Science and Computational Intelligence (DaSCI Institute), Granada, Spain; ^11^Department of Biochemistry, Molecular Biology III and Immunology, University of Granada, Granada, Spain; ^12^Nuclear Medicine Unit, Hospital Universitario Clínico San Cecilio, Granada, Spain; ^13^Angiology and Vascular Surgery Unit, Hospital Universitario Clínico San Cecilio, Granada, Spain; ^14^Department of Medicine, University of Granada, Granada, Spain

**Keywords:** cardiovascular disease, insulin resistance, osteoglycin, type 2 diabetes, vascular smooth muscle cells

## Abstract

Osteoglycin, a fundamental proteoglycan within the vascular extracellular matrix, is expressed in vascular smooth muscle cells (VSMCs). Type 2 diabetes (T2D) is associated with cardiovascular disease (CVD) but the role of osteoglycin in the development of CVD is controversial to date. Therefore, our aims are to determine and compare the level of osteoglycin in T2D patients with/without CVD versus control subjects both at serum and vascular tissue and to analyze in vitro role of osteoglycin in VSMCs under calcified conditions. For this, serum osteoglycin levels were determined by enzyme-linked immunosorbent assay (ELISA) in 117 controls and 129 patients with T2D (46 with CVD and 83 without CVD), revealing a significant increase in patients with T2D compared with controls. Osteoglycin level was not an estimator of CVD but correlated with markers of insulin resistance (triglycerides and triglycerides/high-density lipoprotein cholesterol index) in patients with T2D. At the vascular level, osteoglycin expression was assessed by RT-qPCR and immunohistochemistry, and no significant differences were observed between calcified arteries from patients with T2D and noncalcified arteries from controls. In vitro experiments using VSMCs (mock and overexpressing osteoglycin) under calcifying conditions were performed to analyze the osteoglycin function. The overexpression of osteoglycin in VMSCs under calcifying conditions revealed an increase of cell proliferation without effect on apoptosis and an upregulation of the expression of autotaxin (*ATX*) involved in inflammatory processes. In conclusion, osteoglycin could play a role in glycemic homeostasis, being a potential biomarker of insulin resistance in patients with T2D. Furthermore, osteoglycin could indirectly participate in the development of atherosclerosis through its regulatory effect on *ATX* and by proliferating VSMCs.

**NEW & NOTEWORTHY** This study uncovers an increase of serum osteoglycin levels in patients with type 2 diabetes, which does not appear to be associated with the development of atherosclerosis, but rather with insulin resistance in this population. Overexpression of osteoglycin increased proliferation and upregulated the expression of autotaxin in vascular smooth muscle cells within calcified environments. Osteoglycin could be a biomarker of insulin resistance for type 2 diabetes and could be indirectly involved in the development of atherosclerosis.

## Graphical abstract

**Figure:**
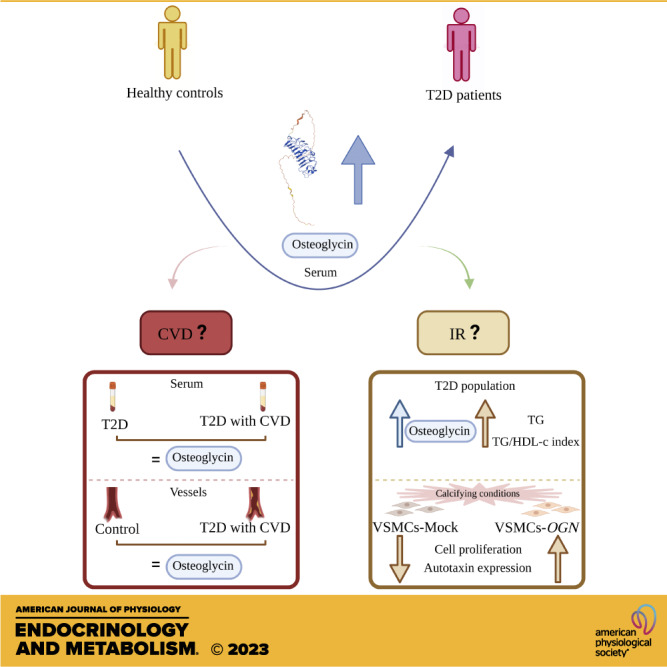

## INTRODUCTION

Type 2 diabetes (T2D) is associated with an increased risk of cardiovascular disease (CVD; 1), affecting ∼35% of this population (2). Atherosclerosis is the main pathological mechanism underlying CVD in T2D, leading mainly to coronary heart disease, cerebrovascular disease, and peripheral arterial disease (3). The development of atherosclerosis is a complex process involving numerous cells, including immune cells, endothelial cells, and vascular smooth muscle cells (VSMCs; 4). There is a strong evidence that phenotypic switching of the VSMCs plays a key role in the development of atherosclerosis (5). Indeed, during disease development, VSMCs proliferate and migrate from the media layer of the arterial wall into the intima layer, contributing to atherosclerotic plaque formation through extracellular matrix (ECM) production and lipid accumulation (5, 6). The ECM contains different proteins, including collagens, elastin, glycoproteins, glycosaminoglycans, and proteoglycans, which provide the structural integrity of the plaques and are involved in several key events, such as cell migration and proliferation, lipoprotein retention, and thrombosis (7).

Osteoglycin, also known as osteoinductive factor or mimecan and encoded by the osteoglycin (*OGN*) gene, is a class III member of the small leucine-rich proteoglycan (SLRP; 8), a distinct group of extracellular proteoglycans, being a basic component of the vascular ECM, expressed mainly by cardiomyocytes, cardiac fibroblasts, and VSMCs (9). Furthermore, this protein is involved both in bone remodeling and in vascular metabolism (10). This indicates that osteoglycin could have a relevant role in vascular pathophysiology; however, few studies have been carried out to elucidate the mechanism of action of this protein at vascular level, which remains unclear to date. Some studies have reported an association between elevated osteoglycin levels in serum with increased cardiovascular risk (10–13) and mortality (14, 15). In this line, it has been reported that reduced osteoglycin levels in patients with complex cardiovascular lesions suggesting a role of osteoglycin in the stabilization of coronary plaque (16). Although most of the studies postulate a detrimental role for osteoglycin. Van Aelst et al. (10) reported that increased osteoglycin expression is essential in the infarct scar, as it promotes proper collagen maturation and protects against cardiac impairment in humans. On the other hand, no association was found between circulating levels of osteoglycin and progression of atherosclerosis in patients with carotid artery plaque (17), and it was shown that osteoglycin deficiency did not affect the progression of atherosclerosis in mice (18). Furthermore, osteoglycin levels were not associated with major adverse cardiovascular, cerebrovascular events, and mortality in T2D patients with chronic kidney disease (CKD; 14).

This study aims to investigate the involvement of osteoglycin in the development of atherosclerosis in humans, which is highly controversial. To research our main objective, we first proposed to quantify osteoglycin levels in serum and vascular tissue samples obtained from patients with T2D, both with and without CVD, as well as from healthy controls. This analysis allowed us to evaluate the association between osteoglycin and atherosclerosis in a clinical context. Second, we conducted experiments using osteoglycin in VSMCs exposed to calcified environments in vitro, to examine the direct impact of this protein on the development of atherosclerosis. By combining clinical and experimental investigations, our study aims to provide comprehensive insights into the role of osteoglycin in the processes of atherosclerosis in humans with T2D.

## MATERIALS AND METHODS

### Study Population

In this cross-sectional study, a total of 246 participants were included: 117 healthy subjects (65 ± 9 yr, 55.6% males) and 129 patients with T2D (65 ± 8 yr, 58.1% males). The healthy subjects were recruited between 2020 and 2022 from the reference population of the Hospital Universitario Clínico San Cecilio of Granada (Spain). The patients with T2D were diagnosed based on the criteria established by the American Diabetes Association (19) and were recruited between 2017 and 2018 from the Endocrinology and Nutrition Unit of the same hospital. Rigorous selection criteria, including Caucasian ethnicity and normal values for blood count and hepatic function, were applied to both groups to ensure the inclusion of individuals suitable for the study. The T2D group was further divided into two subgroups depending on the presence of CVD (*n* = 46) or not (*n* = 83). A statistical power analysis considering the proportion of patients with T2D suffering from CVD in our study population (35.6%) was performed. The scientific literature corroborates this proportion describing a mean prevalence of 32.2% of CVD in patients with T2D worldwide (20, 21). Considering our sample comprises 46 T2D patients with CVD and 83 T2D patients without prevalent CVD our statistical power is >85% (e.g., 87.7%). Calculations were obtained using G*Power (v.3.1.9.7.), using proportions for two independent groups (Fisher’s exact test) and an α significance of 0.05. Both groups of patients with T2D received statin treatment, and specifically the T2D patients with CVD were treated with high-intensity statins. The inclusions criteria for CVD were coronary heart disease (previous myocardial infarction, a diagnosis of stable or unstable angina, or previous coronary revascularization surgery), ischemic cerebrovascular disease (transient ischemic attack or ischemic stroke), or ischemic peripheral arterial disease. Exclusion criteria involved patients with hepatic, gastrointestinal, thyroid, or bone diseases, as well as those with an estimated glomerular filtration rate (eGFR) below 45 mL/min/1.73 m^2^ or receiving treatment with thiazolidinediones.

Vascular tissue samples were obtained from the artery of lower limbs at the Angiology and Vascular Surgery Unit of the Hospital Universitario Clínico San Cecilio of Granada. Calcified vessels were obtained from T2D patients with ischemic diabetic foot with criteria of critical ischemia, who were not suitable candidates for revascularization (primary amputation) or had experienced failed revascularization (secondary amputation; *n* = 6). Noncalcified vessel samples were obtained from healthy subjects who provided informed consent at the Hospital Universitario Clínico San Cecilio of Granada (*n* = 3).

The study adheres to the general ethical principles of the Declaration of Helsinki. Before starting the study, the project was reviewed and approved by the Research Ethics Committee of Granada on April 26, 2017 (Project ID: 0858-N-17). Before participation, informed written consent was provided for each participant. The Biobank of the Andalusian Public Health System at the Hospital Universitario Clínico San Cecilio of Granada managed all the samples used in this study.

### Clinical Evaluation and Biochemical Measurements of Study Population

Baseline height, weight, and waist circumference measurements were obtained from the entire study population using standard procedures. The body mass index (BMI) was calculated using the Quetelet formula: weight (kg)/stature (m^2^). Moreover, for patients with T2D, dyslipidemia was characterized by serum levels of low-density lipoprotein cholesterol (LDL-c) >100 mg/dL, high-density lipoprotein cholesterol (HDL-c) <50 mg/dL, triglycerides (TG) >150 mg/dL, and/or current treatment with lipid-lowering drugs. Systolic and diastolic blood pressures were measured using a standard electronic sphygmomanometer. Hypertension was defined as values equal to or exceeding 140/90 mmHg and/or the use of antihypertensive medication. Alcohol consumption, smoking, and physical activity levels were assessed using the Spanish version of the Rapid Assessment of Physical Activity questionnaire (22). Regarding biochemical measurements, venous blood samples were collected in the morning after an overnight fast, and serum samples were stored at −80°C until analysis, which was carried out at the Clinical Analysis Unit of the Hospital Universitario Clínico San Cecilio of Granada (Spain) of the entire study population. Fasting plasma glucose (FPG), glycated hemoglobin (HbA1c), TG, HDL-c, LDL-c, eGFR, calcium, and phosphorus were measured using standard automated laboratory techniques. The TG/HDL-c index was calculated as an indicator of insulin resistance. eGFR was calculated using the Chronic Kidney Disease Epidemiology Collaboration equation (CKD-EPI; 23). Osteoglycin level was determined by the enzyme-linked immunosorbent assay (ELISA) method, following the manufacturer’s protocols (Cloud Clone Corp.), and precision testing was performed by the determination of intraassay and interassay variances (10% and 12%, respectively).

### Immunohistochemistry and Data Acquisition of Osteoglycin in Vascular Tissue

Formalin-fixed paraffin-embedded biopsy tissues were obtained from the calcified lower limb artery of patients with T2D and noncalcified from control subjects. To enable antigen retrieval, the tissue sections (3 µm) were subjected to high temperature (200°C) incubation in 1× citrate buffer using a steamer machine for 20 min. Then, the tissue sections were deparaffined using a standard protocol involving xylenes and ethanol. Subsequently, the sections were rinsed in phosphate-buffered saline (PBS; 0.01 M, pH 7.4), treated with 3% hydrogen peroxide for 15 min, and rinsed again. A solution containing 3% normal goat serum and 0.1% PBS-tween20 was applied to the sections for 1 h. The slices were exposed overnight at 4°C to an anti-osteoglycin primary antibody (1:200, SC-374463, Santa Cruz). Following a rinse with PBS, the tissue sections were incubated with Goat Anti-Mouse IgG secondary antibody [1:5,000, Goat Anti-Mouse IgG H&L (HRP), No. ab6789, Abcam] at room temperature for 2 h. Both antibodies were diluted in 3% normal goat serum and 0.1% PBS-tween20. The sections were rinsed, then processed using the ABC-kit (Vector Laboratories), and the reaction was visualized using the peroxidase substrate kit DAB (Vector Laboratories). The tissue sections were rinsed, rehydrated with ethanol and xylenes, and cover slipped. For data acquisition of tissue immunohistochemistry, microphotographs were captured using a light microscope, Olympus BX41. Slices containing the regions of interest were identified by Stereo Investigator Software (Bioscience) from coronal sections of the samples (Fig. 4*A*). In each sample, microphotographs of the intima-media and adventitia layers were captured at ×20 magnification (Fig. 4*B*). Image J Software was used to quantify osteoglycin expression. For each microphotograph, the software automatically identified proteins as threshold objects. These objects appeared as black circular dots against a white background and met specific size criteria (ranging from 35 to 150 µm^2^) and circularity values (from 0.35 to 1.00). To ensure consistency across all microphotographs and eliminate potential background noise, we first converted them into 8-bit type images. Then, we adjusted the background by increasing its brightness to 150.0 pixels. The threshold settings for all images were standardized to a range of 0–150.

### RNA Isolation and RT-qPCR

In this study, we performed RNA isolation and reverse transcription-quantitative polymerase chain reaction (RT-qPCR) to measure the expression of osteoglycin in vascular tissue and to assess the efficiency of lentiviral transduction in primary human aortic smooth muscle cells (HAoSMCs). Furthermore, we quantified the expression of various genes under different conditions in HAoSMCs in vitro. To obtain total RNA from vascular tissue, we isolated 23 transversal sections from the calcified lower limb artery of patients with T2D and peripheral artery disease, as well as from the noncalcified lower limb artery of healthy donors. We used Trizol reagent (Thermo Fisher Scientific) and a manual homogenizer for RNA extraction. For cell samples, we used the RNeasy mini kit (QIAGEN) following the manufacturer’s instructions. In both cases, Turbo DNase (Ambion) was used to treat the RNA, and we assessed the RNA concentration and quality using the Qubit Flex Fluorometer (Thermo Fisher Scientific). Only RNA samples with an A260/280 ratio between 1.8 and 2.0 were included for cDNA synthesis, performing reverse transcription with the iScript cDNA synthesis kit (BioRad), according to the manufacturer’s protocol. For qPCR analysis, we used the PowerUp SYBR Green Master Mix in a QuantStudio 7 Flex Real-Time PCR system (Thermo Fisher Scientific). The qPCR protocol was as follows: 95°C 2 min; 40× (95°C 20 s, 65°C 20 s); 65°C to 95°C with an increment of 0.5°C every 4 s. We designed the primers using Clone Manager Suite program (Table 1). To normalize the mRNA data, we used the expression of a constitutive gene. Each real-time PCR reaction was performed in triplicate for each sample. Relative expression of each gene of interest was assessed using the 2^−ΔΔCt^ method (25).

**Table 1.** Primers used in this study

Gene	Sequence (5′–3′)	Amplicon (pb)	Application	Reference
*OGN*		151	Check the efficiency of HAoSMCs transduction. Expression in vascular tissue. Quantify the expression under different conditions in HAoSMCs in vitro.	This study
Forward	CCTCACCTTCCTCTACTTGGACCATA			
Reverse	CCGGATGTAACTGGTGTCATTAGC			
*ATX*		158	Quantify the expression under different conditions in HAoSMCs in vitro.	(24)
Forward	ATGCCTGAATGACTCCACTGTT			
Reverse	AGGACTAAATGTGGCAACTGTG			
*RPL13*		228	Constitutive gene	This study
Forward	CGTAAGATCCGCAGACGTAAGGC			
Reverse	GGACTTGTTCCGCCTCCTCGGAT			

*ATX*, autotaxin; HAoSMCs, primary human aortic smooth muscle cells; *OGN*, osteoglycin; *RPL13*, ribosomal protein L13.

### Cell Cultures

Human embryonic kidney 293 T cells (HEK293T) (ATCC) were cultured in DMEM/F-12 GlutaMAX (Gibco) supplemented with 10% fetal bovine serum (FBS) [NeoBiotech RNase A (Powder)].

HAoSMCs (ATCC) were cultured using vascular cell basal medium (ATCC) supplemented with the VSMC growth kit (ATCC), which consists of various components including recombinant human (rh) fibroblast growth factor, rh insulin, rh epidermal growth factor, l-glutamine, ascorbic acid, and FBS.

Both cell cultures were maintained under standard conditions, which consists of an incubation temperature of 37°C, 5% CO_2_, and a humid atmosphere. Cells were grown to confluence and used for experiments from passages 4–5.

### Second-Generation Lentiviruses and Transduction for Generation of Stable Lines of HAoSMCs

To generate stable lines of HAoSMCs with overexpression of osteoglycin, a second-generation lentiviral packaging system protocol was performed using the following vectors: pVSV-G, which expresses the envelope gene of the VSV-G virus; psPAX2, which expresses the reverse transcriptase gene, protease gene, and gene for assembly of the HIV-1 virus; and the pLVX:*OGN* construct or empty pLVX. All plasmids were obtained from Addgene. HEK293T cells were transfected with a mixture of the above plasmids using polyethyleneimine (Quimigen). The transfected cells were then cultured in DMEM/F-12 GlutaMAX supplemented with 10% FBS under standard conditions for 24 h. Lentivirus particles were harvested, filtered, ultracentrifuged, and resuspended in PBS.

The HAoSMCs were transduced using the lentivirus particles in the presence of polybrene infection reagent (8 mg/mL, Merck) and subsequently selected with hygromycin B (50 mg/mL, Thermo Fisher Scientific). Control cells (mock) were transduced with lentiviruses generated from the empty pLVX vector. The transductions were performed in triplicate, and the cells were cultured under standard conditions. RT-qPCR was performed to test for osteoglycin overexpression in this stable cell line.

### Induction of Calcifying Conditions In Vitro

HAoSMCs that had been transduced with osteoglycin overexpression and mock were seeded on six-well plates at a confluency of 1,000 cells/well. The cells were cultured in a growth medium containing 1.5 mM CaCl_2_ and 10 mM β-glycerophosphate for a maximum of 20 days to stimulate matrix calcification. Throughout the incubation period, the cells were maintained under standard conditions, and the growth medium was changed every 2–3 days.

### Cell Proliferation Assay

Cell proliferation was determined by 3-(4,5-dimethyl-thiazol-2-yl)-2,5-diphenyltetrazolium bromide (MTT) assay. HAoSMCs transduced with osteoglycin overexpression and mock were seeded in a 96-well plate at a density of 250 cells/100 μL/well under calcified conditions. The cells were maintained in standard conditions for 10 days, and the cell proliferation assay was performed every 2 days. At different time points, 10 μL of MTT solution (5 mg/mL) was added to each well, and the plate was incubated for 6 h under standard conditions. Subsequently, 100 μL of lysis buffer (20% SDS in 50% formamide, pH 4.7) was added, and the plate was kept under standard conditions overnight to allow for cell lysis. Cell proliferation was quantified by measuring the optical density (OD) at 570 nm using a spectrophotometer. Four replicates were performed for each condition, and the values were corrected by subtracting the background signal from cell-free media controls.

### Cell Apoptosis Assay

The percentages of cell apoptotic under calcified conditions were analyzed using a FITC annexin V apoptosis detection kit (BD Biosciences). HAoSMCs with osteoglycin overexpression and mock were washed twice with PBS, and a density of 10^5^ cells/100 μL was incubated with annexin V-FITC and propidium iodide for 15 min at room temperature and in darkness for each condition. The samples were analyzed using a BD FACSAria IIIu flow cytometer (Becton Dickinson, BD Biosciences). To calculate the percentage of apoptosis, the sum of early apoptotic cells (Annexin-FITC+/PI−) and late apoptotic cells (Annexin-FITC+/PI+) was determined. Each condition was performed in duplicate.

### Statistical Analysis

For statistical analyses, SPSS 28.0 (IBM Corp.) and GraphPad Prism v7.03 (GraphPad Software) were used.

Serum data were presented as means ± standard deviation (SD) or median with interquartile range (IQR) depending on normality. Categorical variables were expressed as percentages. Normality of continuous variables was assessed using the Kolmogorov–Smirnov test. Group comparisons for normally distributed variables were performed using unpaired Student’s *t* test, whereas the Mann–Whitney *U* test was used for nonnormally distributed variables. One factor analysis of variance single factor (One-Factor ANOVA) was used to compare several groups. Covariate-adjusted group comparisons were conducted using univariate analysis of covariance (ANCOVA). Categorical variables were compared using the χ^2^ test. Correlations between continuous variables were assessed using Spearman’s correlation coefficients. Multiple linear regression was utilized to identify independent variables associated with osteoglycin levels in serum, and the data were expressed as B; 95% confidence interval (CI; lower limit/upper limit). Multiple logistic regression was performed to determine if osteoglycin was an independent predictor of CVD. Statistical significance was set at *P* < 0.05 (two tailed) and *P* < 0.10 for multiple linear and logistic regression analysis.

Immunohistochemistry and RT-qPCR were performed on vascular tissue to assess osteoglycin expression. Data were presented as means ± SD, and the differences between different groups and tissue layers were compared using unpaired Student’s *t* test, providing a statistical assessment of the significance of the observed variations. In vitro analysis of HAoSMCs presented data as mean ± SD. Group differences were assessed using unpaired Student’s *t* test to determine significance of observed variations between groups.

## RESULTS

### Characteristics of the Study Population

Table 2 summarizes the baseline characteristics of the entire population consisting of healthy subjects and patients with T2D. Both groups were comparable in age and sex. As expected, patients with T2D showed a significantly worse metabolic profile in terms of BMI, waist circumference, FPG, HbA1c, and lipid profile. In addition, T2D group showed a significant increase in serum osteoglycin levels compared with control group (*P* < 0.001).

**Table 2.** Comparison of baseline characteristics between the control and T2D groups

Baseline Characteristics	Control	T2D	*P*
Men/women, *n*	65/52	75/54	0.683
Age, yr	65 ± 9	65 ± 8	0.352
Body weight, kg	74.6 ± 14.2	86.3 ± 14.3	<0.001*
Height, cm	163 ± 0.1	165 ± 0.09	0.111
BMI, kg/m^2^	27.9 ± 4.5	31.7 ± 4.5	<0.001*
Waist circumference, cm	96.9 ± 10.6	105.9 ± 10.4	<0.001*
FPG, mg/dL	91 (84–99)	143 (110–176)	<0.001*
HbA1c, %	5.6 (5.4–5.8)	7.6 (7–8.6)	<0.001*
HDL-c, mg/dL	54 ± 12	46 ± 11	<0.001*
LDL-c, mg/dL	116 ± 32	93 ± 41	<0.001*
TG, mg/dL	102 (77–144)	139 (99–196)	<0.001*
TG/HDL-c index	2 (1.3–3)	3.3 (2.3–4.5)	<0.001*
eGFR, mL/min/1.73 m^2^	87.2 (74.5–94.5)	87.2 (74.7–97.5)	0.585
Calcium, mg/dL	9.7 (9.5–10.1)	9.7 (9.5–9.9)	0.401
Phosphorous, mg/dL	3.2 (2.9–3.5)	3.3 (2.9–3.6)	0.293
Osteoglycin, pg/mL	905 (776–1,155)	1802 (1,402–2,444)	<0.001*

Data for continuous and normally distributed variables are presented as means ± standard deviation. Data for continuous variables not normally distributed are presented as median followed by interquartile range in brackets. Data for categorical variables are presented as percentages. Student’s *t* test and Mann–Whitney *U* test were used for comparisons of continuous and normally or not normally distributed variables, respectively, between groups. χ^2^ test was used for comparison of categorical variables between groups. BMI, body mass index; eGFR, estimated glomerular filtration rate; FPG, fasting plasma glucose; HbA1c, glycated hemoglobin; HDL-c, high-density lipoprotein cholesterol; LDL-c, low density lipoprotein cholesterol; T2D, type 2 diabetes; TG, triglycerides; TG/HDL-c index, triglycerides/high-density lipoprotein cholesterol index. *P < 0.05 between groups.

When the patients with T2D were divided according to the presence of CVD, the groups were comparable in most of the variables. The groups differed in sex, age, and various factors defining CVD risk, such as blood pressure, lipid profile, and eGFR. No significant differences were found between the groups in serum osteoglycin levels (Table 3).

**Table 3.** Intergroup comparison for patients with T2D according to the presence of CVD

	T2D without CVD	T2D with CVD	*P*
Men/women, *n*	39/44	36/10	<0.001*
Age, yr	64 ± 7.6	67 ± 7.4	0.017*
*Clinical Evaluation*			
Body weight, kg	87.1 ± 14	84.9 ± 15	0.204
Height, cm	164 ± 0.09	166 ± 0.09	0.185
BMI, kg/m^2^	32.2 ± 4.6	30.8 ± 4.29	0.043*
Waist circumference, cm	106.6 ± 10.6	104.3 ± 10	0.133
Diabetes duration, yr	13 ± 8.6	17 ± 10.2	0.011*
Dyslipidemia, %	83.1	97.8	0.013*
Systolic blood pressure, mmHg	136.2 ± 18	135.3 ± 16.5	0.392
Diastolic blood pressure, mmHg	81.3 ± 9.4	75.5 ± 12.3	0.002*
Hypertension, %	79.5	95.7	0.013*
Coronary heart disease, %		56.5	<0.001*
Cerebrovascular disease, %		23.9	<0.001*
Peripheral artery disease, %		37.0	<0.001*
Smoker or ex-smoker, %	41.5	54.3	0.161
Alcohol consumption, %	15.9	17.4	0.822
Sedentarism, %	11.6	22.2	0.150
*Current Medication Use*			
Insulin, %	68.7	78.3	0.245
Oral antidiabetic drugs, %	31.3	21.7	0.245
*Biochemical Measurements*			
FPG, mg/dL	147.9 ± 48.8	153.9 ± 57.6	0.266
HbA1c, %	7.9 ± 1.3	7.9 ± 1.6	0.463
HDL-c, mg/dL	47 ± 11	43 ± 11	0.029*
LDL-c, mg/dL	101 ± 42	79 ± 36	0.002*
TG, mg/dL	139 (110–205)	142.5 (91–186)	0.192
TG/HDL-c index	3.1 (2.3–4.5)	3.4 (2.2–4.8)	0.906
eGFR, mL/min/1.73 m^2^	86.7 ± 18.3	79.1 ± 19.4	0.014*
Calcium, mg/dL	9.8 (9.5–9.9)	9.6 (9.3–9.8)	0.012*
Phosphorous, mg/dL	3.3 (3–3.7)	3.3 (2.9–3.6)	0.492
Osteoglycin, pg/mL	1,788 (1,474–2,331)	1,866 (1,377–2,861)	0.363

Data for continuous and normally distributed variables are presented as means ± standard deviation. Data for continuous variables not normally distributed, are presented as median followed by interquartile range in brackets. Data for categorical variables are presented as percentages. Student’s *t* test and Mann–Whitney *U* test were used for comparisons of continuous and normally or not normally distributed variables, respectively, between groups. χ^2^ test was used for comparison of categorical variables between groups. BMI, body mass index; CVD, cardiovascular disease; eGFR, estimated glomerular filtration rate; FPG, fasting plasma glucose; HbA1c, glycated hemoglobin; HDL-c, high-density lipoprotein cholesterol; LDL-c, low density lipoprotein cholesterol; TG, triglycerides; TG/HDL-c index, triglycerides/high-density lipoprotein cholesterol index; T2D, type 2 diabetes. *P < 0.05 between groups.

### Influence of Diabetes Status, Sex, and CVD on Serum Osteoglycin Levels

**Figure 1.**
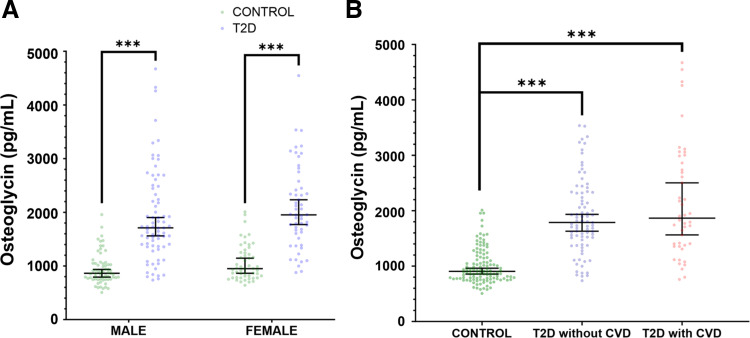
Serum osteoglycin level of the study population. *A*: serum osteoglycin level in male and females for both control (65/52) and T2D groups (75/54). The dot-plot represent median (95% confidence interval) and *P* values between sexes were performed using the Mann–Whitney *U* test. ***P < 0.001. *B*: dot-plot of serum osteoglycin levels in controls (*n* = 117), T2D patients without CVD (*n* = 46), and T2D patients with CVD (*n* = 83), adjusting by age and sex. Dot-plot represent median (95% confidence interval) and *P* values between the different were performed by ANCOVA. ***P < 0.001. CVD, cardiovascular disease; T2D, type 2 diabetes.

Osteoglycin levels in serum were significantly increased in patients with T2D (*n* = 129, 58.1% males) compared with healthy subjects (*n* = 117, 55.6% males) [1,802 (1,403–2,444) pg/mL vs. 905 (776–1,155) pg/mL, *P* < 0.001]. When patients with T2D and healthy subjects were further divided according to sex, the significative differences in osteoglycin levels remained for both males [patients with T2D: 1,079 (1,366–2,444) pg/mL vs. healthy subjects: 864 (756–1,047) pg/mL, *P* < 0.001], and females [patients with T2D: 1,953 (1,606–2,449) pg/mL vs. healthy subjects: 950 (798–1,247) pg/mL, *P* < 0.001]. Moreover, no significant differences in serum osteoglycin levels were found between males and females in the T2D group [1,079 (1,366–2,444) pg/mL vs. 1,953 (1,606–2,449) pg/mL, *P* = 0.082] and the control group [864 (756–1,047) pg/mL vs. 950 (798–1,247) pg/mL, *P* = 0.060; Fig. 1*A*]. Furthermore, when patients with T2D were divided according to the presence of CVD no significant differences were observed between the groups [1,866 (1,377–2,861) pg/mL vs. 1,788 (1,474–2,331) pg/mL, *P* = 0.363]. In addition, serum osteoglycin differences after adjusting by age and sex were only observable between the control and T2D groups (*P* < 0.001) regardless of the presence of CVD (*P* = 0.374; Fig. 1*B*). There were also no differences in serum osteoglycin levels according to the presence of major CVDs in the T2D group [coronary artery disease (*P* = 0.837); ischemic cerebrovascular disease (*P* = 0.616); and peripheral artery disease (*P* = 0.241)]. These findings suggest that serum osteoglycin levels are not tightly involved in CVD in the population with T2D.

### Determinants of Serum Osteoglycin Levels in the Patients with T2D

**Figure 2.**
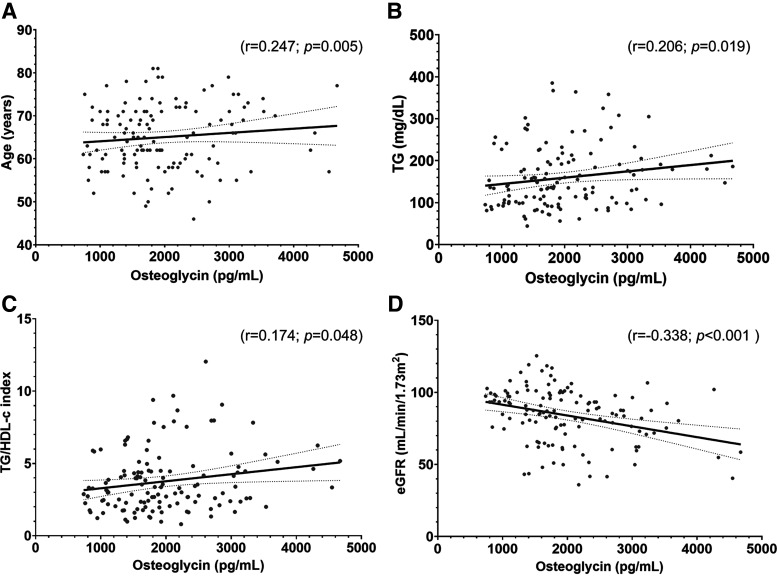
Scatter plots showing the correlation between serum osteoglycin (pg/mL) and: age (years; *A*), TG (mg/dL; *B*), TG/HDL-c index (*C*), and eGFR (mL/min/1.73 m^2^; *D*) in patients with T2D (*n* = 129). The *P* values between the different associations were performed by Spearman’s correlation coefficients (showing *P* < 0.05 in each scatter plot). eGFR, estimated glomerular filtration rate; TG, triglycerides; TG/HDL-c index, triglycerides/high-density lipoprotein cholesterol index; T2D, type 2 diabetes.

A positive correlation between the serum osteoglycin levels and variables such as age (*r* = 0.247; *P* = 0.005), TG (*r* = 0.206; *P* = 0.019), and TG/HDL-c index (*r* = 0.174; *P* = 0.048) was observed in patients with T2D. In addition, a negative correlation was found between serum osteoglycin levels and eGFR (*r* = −0.338; *P* < 0.001; Fig. 2).

**Figure 3.**
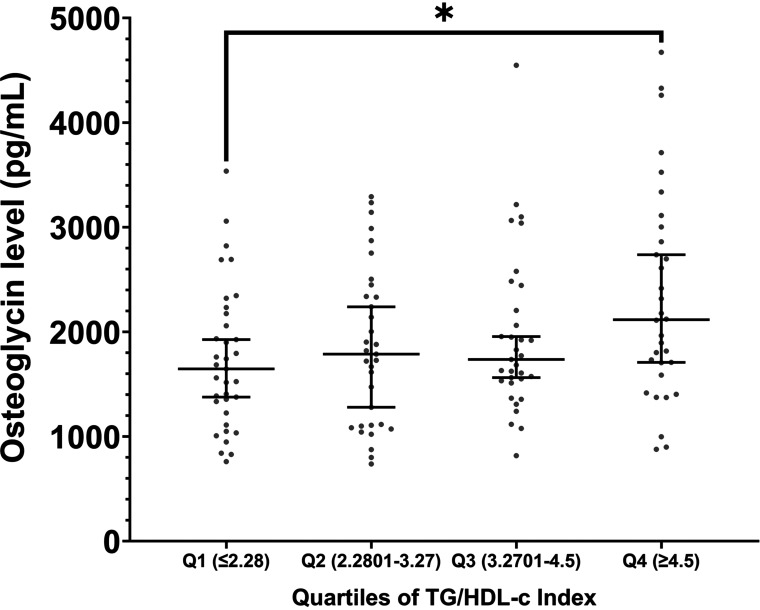
Comparison of circulating osteoglycin levels in the different quartiles (Q) of the TG/HDL-c index in patients with T2D. The dot-plot represents the median of the osteoglycin serum levels and the 95% confidence interval within each Q of TG/HDL-c index (Q1, *n* = 33; Q2, *n* = 32; Q3, *n* = 32; Q4, *n* = 32). The *P* values between quartiles were performed by one-factor ANOVA. *P < 0.005. Q, quartile; TG/HDL-c index, triglycerides/high-density lipoprotein cholesterol index.

In addition, our study showed that circulating osteoglycin levels in patients with T2D increased stepwise from the lowest quartile to the highest quartile of TG/HDL-c index, with significant differences between *quartile 1* and *quartile 4* (*P* = 0.039; Fig. 3), suggesting that osteoglycin is highly related to this index.

To examine the factors influencing osteoglycin levels, a multiple linear regression analysis model was performed. The model included variables identified as being associated with osteoglycin in previous bivariate analysis, such as age, TG, TG/HDL-c index, and eGFR. In addition, sex, current medication, and the presence of CVD were included as independent variables in the analysis. The results showed that the variables independently associated with the serum osteoglycin level were age (*B* = 0.232; 95% CI [6.3/44.0]; *P* = 0.009), TG (*B* = 0.217; 95% CI [0.6/4.3]; *P* = 0.009), and eGFR (*B* = −0.251; 95% CI [−18.4/−3.4]; *P* = 0.005).

### Analysis of the Relation between Serum Osteoglycin Level and CVD Risk in Patients with T2D

Logistic regression modeling was performed to assess the variables related to CVD risk in population with T2D. The independent variables included in the model were age, sex, dyslipidemia, hypertension, years of diabetes duration, TG/HDL-c index, eGFR, tobacco use, and sedentarism, in addition to serum osteoglycin level. We showed that the independent variables associated with CVD risk in patients with T2D were sex (OR = 0.228; 95% CI [0.088/0.591]; *P* = 0.002), dyslipidemia (OR = 0.118; 95% CI [0.013/1.099]; *P* = 0.061), hypertension (OR = 0.217; 95% CI [0.043/1.092]; *P* = 0.064), and years of diabetes duration (OR = 1.048; 95% CI [0.993/1.105]; *P* = 0.086). These results revealed that circulating osteoglycin level does not appear to be a predictor of CVD risk in population with T2D.

### Osteoglycin Expression Level in Vascular Tissue

**Figure 4.**
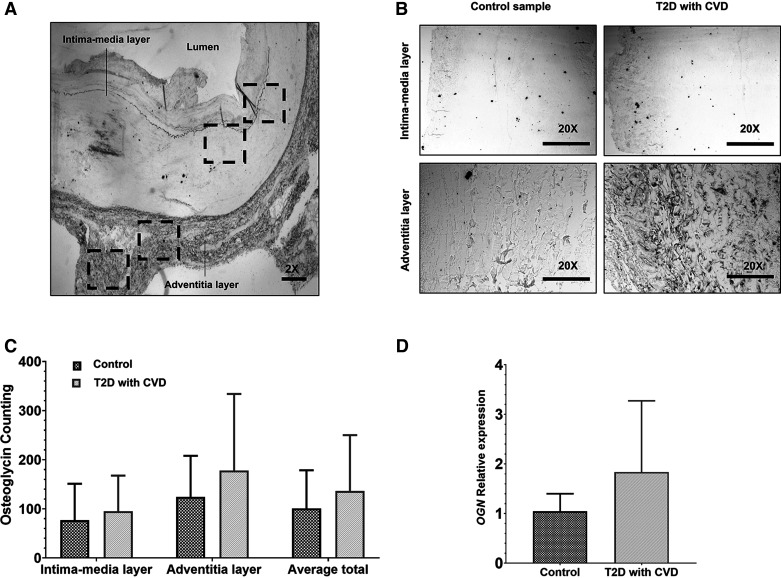
Osteoglycin expression at vascular tissue, specifically in calcified lower limb arteries of patients with T2D (*n* = 6) and noncalcified arteries of control subjects (*n* = 3). *A*: schematics of the microphotographs (×2) of the artery sections used for osteoglycin immunohistochemistry. The microphotographs were systematically captured following a medio-lateral axis to cover the intima-media and the adventitia layer of the artery. The dashed boxes represent the schematic location of the microphotographs captured for both layers. *B*: representative microphotographs obtained at ×20 magnification of the artery for the intima-media layer (*top*) and the adventitia layer of the artery (*bottom*) for control and T2D patients with CVD groups. *C*: counting of osteoglycin labeled proteins in the intima-media and the adventitia layer of the artery and the total average for control and T2D with CVD groups, was performed by immunohistochemistry. *D*: evaluation of osteoglycin relative expression in both groups using the 2^−ΔΔCt^ method (fold-change), was performed by RT-qPCR. All data are presented as the means ± standard error and Student’s *t* test was used for the comparison. CVD, cardiovascular disease; *OGN*, osteoglycin; T2D, type 2 diabetes.

Immunohistochemistry and RT-qPCR were performed on calcified lower limb arteries of patients with T2D (*n* = 6) and noncalcified arteries of control subjects (*n* = 3). Analysis of the total average of osteoglycin-positive cells using immunohistochemistry revealed no significant differences in osteoglycin expression in calcified vessels from patients with T2D compared with noncalcified vessels from control subjects (136.5 ± 46.3 vs. 100.8 ± 44.9, *P* = 0.321). When examining the location of osteoglycin-positive cells, no significant differences were observed between the intima-media layer and the adventitial layer in both patients with T2D (95 ± 29.7 vs. 178 ± 63.6; *P* = 0.132) and control groups (77 ± 42.7 vs. 124 ± 48.1; *P* = 0.251; Fig. 4*C*). In addition, no change in osteoglycin mRNA regulation was found between calcified lower limb arteries of patients with T2D and noncalcified lower limb arteries of control subjects (1.37-fold change; *P* = 0.288; Fig. 4*D*). These results indicate that there is no variation in the expression of osteoglycin in vascular tissue associated with the pathogenesis of vascular calcification in patients with T2D.

### Effect of Osteoglycin Overexpression on Mechanisms Involved in Calcification in HAoSMCs

To examine the influence of osteoglycin on HAoSMCs in a calcified condition, we used a second-generation lentiviral packaging system to establish stable overexpression of osteoglycin in vitro. The effectiveness of osteoglycin overexpression in HAoSMCs was confirmed through RT-qPCR. Remarkably, HAoSMCs transduced with *OGN* gene exhibited a significant 1,364-fold increase in osteoglycin mRNA levels compared with the mock group, indicating successful and robust osteoglycin overexpression (*P* < 0.001) under the same experimental conditions. Furthermore, RT-qPCR was also used to check the impact of osteoglycin overexpression on the regulation of the autotaxin (*ATX*) gene, which encodes the ATX protein, also called ectonucleotide pyrophosphatase/phosphodiesterase family member 2 (*ENPP2*), a protein involved in inflammatory processes. The results revealed that HAoSMCs with osteoglycin overexpression exhibited a significant upregulation of *ATX* compared with mock (16.9-fold; *P* < 0.001).

**Figure 5.**
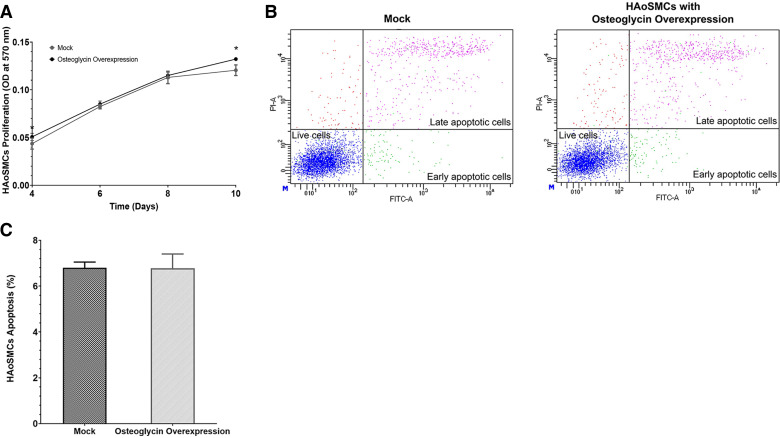
Effect of osteoglycin overexpression on proliferation and apoptosis in HAoSMCs under calcified conditions. *A*: analysis of proliferation in mock compared with HAoSMCs with osteoglycin overexpression (*n* = 4 biological replicates in each time/group). 3-(4,5-Dimethylthiazol-2-yl)-2,5-diphenyltetrazolium bromide (MTT) assay was performed for proliferation, and each result was expressed as OD at 570 nm. Raw data of apoptosis analysis by flow cytometry in mock (*left*) and HAoSMCs with osteoglycin overexpression (*right*) (*B*) and determination of the percentage of apoptosis between both cultures (*C*). To calculate the percentage of apoptosis, the sum of early apoptotic cells (Annexin-FITC+/PI−) and late apoptotic cells (Annexin-FITC+/PI+) was determined (*n* = 3 biological replicates/group and *n* = 2 technical replicates/biological replicates). All data represent as the means ± standard deviation of experiments performed and the *P* values between groups were determined by the unpaired Student’s *t* test. *P < 0.05 vs. mock. HAoSMCs, primary human aortic smooth muscle cells; OD, optical density.

The effect of osteoglycin overexpression on the proliferation and survival of HAoSMCs was evaluated in calcified conditions. The MTT assay was performed to determine how osteoglycin overexpression affected the proliferation of HAoSMCs. The results revealed a slight significant increase in the proliferation rate of HAoSMCs with osteoglycin overexpression compared with the mock group. Specifically, at 4 days, there was a 16% increased (*P* = 0.042), at 6 days a 3% increased (*P* = 0.122), at 8 days a 2% increased (*P* = 0.297), and at 10 days an 8% increased (*P* = 0.003; Fig. 5*A*). Furthermore, flow cytometry was used to investigate apoptosis-induced cell death after annexin V and propidium iodide staining (Fig. 5*B*). No discernible differences in the percentage of apoptosis between mock compared with HAoSMCs with osteoglycin overexpression were observed (6.8 ± 0.3%, vs. 6.8 ± 0.6%, *P* = 0.476; Fig. 5*C*).

## DISCUSSION

Our study reported for the first time an increase in serum osteoglycin levels in patients with T2D compared with nondiabetic controls. This elevation does not appear to be associated with the development of atherosclerosis in this population. However, there is a potential relationship of osteoglycin with markers of insulin resistance.

The function of osteoglycin at the vascular level is controversial and not well understood. Some studies have reported a close relationship between osteoglycin levels and the risk of suffering CVD showing an association between an increase of osteoglycin in serum and patients with coronary artery disease (12, 16), as well as, increased arterial stiffness in hypertensive patients (13). Within this line, it has been suggested that osteoglycin could be used as a prognostic biomarker in patients with coronary artery disease (15). In addition, this protein may act as a predictor for the incidence of cardiovascular events in patients with acute coronary syndrome or CKD (14, 15), as well as for mortality in patients with carotid artery disease (11). However, some studies point in the opposite direction, describing the beneficial role for osteoglycin against cardiac impairment in humans (10). Despite this evidence, our results found no association between serum osteoglycin levels with the presence of CVD overall nor with the presence of specific manifestations of CVD in patients with T2D. In addition, our statistical results did not position the presence of CVD as a variable influencing osteoglycin levels. Likewise, osteoglycin was not an independent estimator of CVD risk in patients with T2D. On the other hand, our results also revealed no significant differences in osteoglycin expression in calcified vessels from patients with T2D, both in the intima-media and adventitia layers, compared with noncalcified vessels from control subjects. This indicates that the elevation of serum osteoglycin in patients with T2D relative to controls is not derived from vascular expression. By contrast, a previous in situ hybridization study in human coronary arteries showed that osteoglycin mRNA was expressed by normal medial VSMCs but was downregulated in a subset of intimal VSMCs (9). Furthermore, an upregulation of osteoglycin in the thick neointima and in the front edge of migrating VSMCs has been described in rabbits with atherosclerotic lesions (26). Consistently with our results, some studies support the noncorrelation between circulating osteoglycin levels with major adverse cardiovascular, cerebrovascular events, and mortality in T2D patients with CKD (14) nor with atherosclerosis development in patients with carotid artery plaque (17) or animal models (18).

Based on this, osteoglycin does not appear to be a key determinant in the pathogenesis of atherosclerosis in patients with T2D directly, although it may play an indirect role through its involvement in insulin resistance. The elevation of circulating osteoglycin levels in patients with T2D compared with controls could be explained partially by influencing variables such as age, circulating TG level, TG/HDL-c index, and eGFR. Osteoglycin is a hormone highly expressed in adipose tissue and secreted into the circulation, in both mice (27, 28) and humans with obesity (29). Our results showed a positive correlation between serum osteoglycin levels and the TG level and TG/HDL-c index which are associated with glucose disturbances (30). Specifically, the TG/HDL-c index is used as a predictor of insulin resistance and CVD risk (31). Our results showed a positive correlation between serum osteoglycin levels and the TG/HDL-c index in patients with T2D observing an increase in circulating osteoglycin levels according to the TG/HDL-c index quartiles. Thus, the T2D patients with higher insulin resistance (fourth quartile of TG/HDL-C index) are those who showed higher levels circulating osteoglycin levels. This finding suggests that osteoglycin could be a mediator of insulin resistance. We propose that the osteoglycin-mediated insulin resistance may be related to the ATX pathway. Our in vitro experimental analysis showed for first time that an osteoglycin overexpression leads to an upregulation of *ATX* encoding for ATX, which is a protein with lysophospholipase D activity catalyzing the hydrolysis of lysophospholipids into lysophosphatidic acid (LPA). Studies have demonstrated that the ATX/LPA axis is involved in T2D, insulin resistance, and obesity (32, 33). Accordingly, it has been reported an increase in ATX expression among patients with T2D, suggesting a potential involvement of this protein in the development of the disease (34), whose expression could be influenced by alterations in glucose homeostasis affecting insulin and glucose levels in these patients with T2D (32). In addition, the ATX/LPA axis has been involved in CVD, particularly atherosclerosis (33), by enhancing the penetration of blood monocytes into the subendothelial space of vascular tissue, endothelial dysfunction, and proliferation of VSMCs (35). An increase in the proliferation of VSMCs implies an increase in collagen synthesis promoting artery stiffening and the formation of atherosclerotic plaque (36, 37). Regarding cell apoptosis, there is evidence supporting that a high rate of VSMCs apoptosis can promote vascular calcification (38) and contribute to plaque instability (39) favoring the atherosclerotic process. Few studies have attributed to osteoglycin a reducing effect on the proliferation of VMSCs (40, 41). However, our experimental results showed an increase in the proliferation rate of HAoSMCs associated with osteoglycin overexpression without influence on HAoSMCs apoptosis under calcifying conditions. These findings suggest that osteoglycin plays a key role mainly in insulin resistance and could indirectly participate in the development of atherosclerotic process through activation of the ATX/LPA pathway and proliferation of VSMCs within the population with T2D. However, future studies are needed to fully determine the molecular mechanism of osteoglycin in this regard.

On the other hand, it is well known that cardiovascular and renal alterations are closely related in patients with T2D (42). Some studies have reported higher osteoglycin levels in serum in T2D patients with nephropathy (43). Consistently, our previous study reported increased serum osteoglycin levels associated with mild kidney function impairment in patients with T2D compared with healthy subjects proposing osteoglycin as an early biomarker of kidney impairment (44). In agreement, our results have shown a negative correlation between circulating osteoglycin levels and eGFR in patients with T2D. In contrast, Wei et al. (45) showed that low serum osteoglycin levels were an independent diagnostic marker of diabetic nephropathy with microalbuminuria in patients with T2D (45). This discrepancy may be due to differences in patient populations regarding to age, comorbidities, and variability in the duration of the observational period.

Our study provides valuable data, although it also has some limitations. The cross-sectional design prevents us from establishing a cause-effect relationship, offering only associations between variables. In addition, our study population included only Caucasian individuals from a specific area, and the use of antihyperlipidemic, antihypertensive, and antidiabetic drugs may influence the clinical results. Another limitation is the small number of vascular tissue samples obtained from both controls and T2D patients with CVD. This scarcity is mainly due to the difficulties in acquiring such samples, especially for healthy controls. As a consequence, it is imperative to interpret these results with caution, underscoring the need for future investigations to further validate and expand upon our findings. Despite these limitations, our research presents several strengths such as comprehensive evaluation of osteoglycin at both the clinical level (serum and vascular tissue) and the basic level (in vitro assays) within the same study. Moreover, we meticulously assessed various clinical, anthropometric, and biochemical parameters, covering all the variables that could influence cardiovascular risk, integrating these findings with experimental results. Furthermore, we used rigorous statistical analyses to ensure the reliability of our conclusions. Overall, while acknowledging the limitations, our study contributes significantly to the understanding the role of osteoglycin in the context of atherosclerosis and T2D.

In conclusion, our study describes for the first time that patients with T2D present increased serum osteoglycin levels compared with nondiabetic controls, although we found no evidence to support a relevant role for this protein in the development of atherosclerosis in this population. We demonstrated that osteoglycin plays a key role in glycemic homeostasis and could be a potential biomarker of insulin resistance in patients with T2D. Furthermore, this protein could indirectly participate in the development of atherosclerosis through its regulatory effect on other genes involved in inflammation, such as *ATX* and by VSMCs proliferation. Although further research is necessary to elucidate the intricate pathways in which osteoglycin is involved, these results open the door for the study of osteoglycin as a potential therapeutic target in T2D.

## DATA AVAILABILITY

The data sets generated and/or analyzed during the current study are available from the corresponding author on reasonable request.

## GRANTS

This work was supported by Junta de Andalucía Grant PI0268-2019 and Institute of Health Carlos III Grants PI18-00803 and PI18-01235, cofunded by the European Regional Development Fund (FEDER). Furthermore, it was supported by CIBER Frailty and Healthy Aging (CIBERFES) of the Institute of Health Carlos III. In addition, S.G.-S. is funded by predoctoral fellowship (FI19/00118), and C.G.-F. and B.G.-F. are funded by postdoctoral fellowships from the Institute of Health Carlos III (CD20/00022; CP22/00022, respectively).

## DISCLOSURES

No conflicts of interest, financial or otherwise, are declared by the authors.

## AUTHOR CONTRIBUTIONS

S.G.-S., B.G.-F., L.M.-H., E.M.-A., S.L.-A., C.G.-F., and M.M.-T. conceived and designed research; S.G.-S., B.G.-F., J.L., R.S.-d.l.T., and C.G.-F. performed experiments; S.G.-S., B.G.-F., L.M.-H., F.A.-V., C.G.-F., and M.M.-T. analyzed data; S.G.-S., B.G.-F., F.A.-V., C.G.-F., and M.M.-T. interpreted results of experiments; S.G.-S. prepared figures; S.G.-S., B.G.-F., L.M.-H., C.G.-F., and M.M.-T. drafted manuscript; S.G.-S., B.G.-F., C.G.-F., and M.M.-T. edited and revised manuscript; S.G.-S., B.G.-F., L.M.-H., J.L., F.A.-V., R.S.-d.l.T., E.M.-A., S.L.-A., C.G.-F., and M.M.-T. approved final version of manuscript.
